# Reversibly-sealable microfluidic platform for multi-molecule gradient delivery to large adherent cell cultures

**DOI:** 10.1007/s10544-026-00831-z

**Published:** 2026-06-17

**Authors:** Julia Radzio, Łukasz Suprewicz, Da Kuang, Alexander Karpowicz, Paul A. Janmey, Jai-Yoon Sul, David A. Issadore, James H. Eberwine, Paulo E. Arratia

**Affiliations:** 1 Department of Mechanical Engineering and Applied Mechanics, School of Engineering and Applied Science, University of Pennsylvania, Philadelphia, PA USA; 2 Department of Physiology and Institute for Medicine and Engineering, Perelman School of Medicine, University of Pennsylvania, Philadelphia, PA USA; 3 Department of Systems Pharmacology and Translational Therapeutics, Perelman School of Medicine, University of Pennsylvania, Philadelphia, PA USA; 4 Department of Bioengineering, School of Engineering and Applied Science, University of Pennsylvania, Philadelphia, PA USA

**Keywords:** Microfluidics, Reversibly-sealable, Gradient generation, Adherent cells

## Abstract

Spatial manipulation of flow gradients and chemical microenvironments is essential for understanding fundamental biological mechanisms and investigating therapeutic responses in adherent cells. Convection-dominated gradient generators in microfluidic devices enable tunable chemical and shear stress gradients across large cell culture areas. However, most concentration generators are irreversibly sealed and operate in a narrow range of shear stresses, which restricts access to the cells after treatment and the physiological relevance of the flow conditions. Here, we present a reversibly sealable microfluidic platform that enables spatiotemporally controlled delivery of multiple small molecules to mammalian cells grown on large glass coverslips. Our device generates a relatively wide range of shear stresses and robust, spatially predictable chemical gradients across centimeter-scale areas and provides optical access compatible with live-cell imaging; it operates in the Stokes and laminar flow regimes. A mechanical sandwich clamp enables leak-free perfusion into the cell culture chamber and access to the cells after treatment. We experimentally and numerically demonstrate the ability to modulate the amount of mixing between co-flowing streams of small molecules. We verify the uptake of fluorophores across a monolayer of cells and assess their viability after perfusion and removal from the device. This platform provides a versatile and reusable approach for studying cellular responses to microenvironmental gradients in varied physiologically relevant shear stress conditions.

**Supplementary Information:**

The online version contains supplementary material available at https://doi.org/10.1007/s10544-026-00831-z.

## Introduction

*In vivo*, adherent cells are rarely exposed to uniform chemical, mechanical, and structural environments (Chen et al. 2020). Instead, they continuously experience spatial and temporal gradients of mechanical stresses such as matrix stiffnesses and shear forces, and soluble factors, including nutrients, oxygen, metabolites, cytokines, growth factors, and therapeutic agents (Discher et al. 2009; Wouters and Koritzinsky 2008). These gradients arise from transport limitations, fluid flow, tissue architecture, and cellular consumption or secretion, and they play a central role in regulating cell fate, function, and responsiveness to external stimuli (Suh et al. 2024).

Physiological examples of microenvironmental gradients are prevalent. In tissues, diffusion-limited transport establishes gradients of oxygen and metabolites that regulate proliferation, differentiation, and survival (Carreau et al. 2011; Li et al. 2025). In the kidney, steep gradients of osmolytes, ions, and signaling molecules with resolutions on the order of several millimeters along the nephron are essential for filtration, reabsorption, and concentration of urine, while tubular epithelial cells respond to highly localized chemical cues at the cell–fluid interface (Layton and Layton 2019; Alberts et al. 2002; Knepper et al. 2003). Similarly, endothelial cells are exposed to gradients shaped by flow and transendothelial transport, influencing inflammation, permeability, and drug uptake (Józkowiak et al. 2025). At the vascular interface, steep biochemical gradients form over micrometer-length scales; oxygen can fall by 0.7 mmHg/µm, while nitric oxide can decrease by tens to hundreds of nanomolar within 1–50 µm (Tsai et al. 1998; Nayak et al. 2024. Simultaneously, flow-induced shear stresses as low as 0.1 Pa up to 9.5 Pa can influence the fate of various cell types (Espina et al. 2023; Koutsiaris et al. 2007). For example, neuronal cells experience minimal effects on viability and differentiation at shear stresses below 0.5 Pa, while long-term exposure above 1 Pa significantly decreases viability (Liu et al. 2013). In contrast, endothelial cells respond favorably to shear stresses between 0.3 and 2 Pa, which promote vascular barrier function (Polacheck et al. 2017) and wound healing (van der Meer et al. 2010; Albuquerque et al. 2000).

Microenvironmental gradients are also highly relevant during systemic therapeutic interventions. Following intravenous administration, drugs do not distribute homogeneously at the cellular level (Thurber et al. 2013). Instead, concentration gradients form across tissues, vessel walls, and extracellular matrices, leading to spatially heterogeneous drug exposure (Minchinton and Tannock 2006). In chemotherapy, for example, tumor-adjacent cells may encounter drug concentrations that vary by orders of magnitude over micrometer-to-millimeter length scales due to vascular heterogeneity, interstitial pressure, and diffusion barriers (Jain 2012; Wojtkowiak et al. 2011). Such gradients critically influence therapeutic efficacy, resistance development, and off-target toxicity, yet remain difficult to reproduce in conventional cell culture systems.

Despite their physiological and clinical importance, microenvironmental gradients are largely absent in standard *in vitro* assays (Keenan and Folch 2007). Conventional *in vitro* systems, such as multi-well plates and Petri dishes, have been used for investigating cellular responses to small molecules and biologics. However, these assays fail to recapitulate the complex microenvironmental and physiological conditions present *in vivo*, including fluid flow, contributing to poor efficacy in preclinical trials and high drug attrition rates (Kuo and Barrett 2024; Jain 2012).

Microfluidic devices offer an alternative method for *in vitro* cell-based screening (El-Ali et al. 2006; Chen et al. 2020). In microscale geometries, low-Reynolds number hydrodynamics (Squires and Quake 2005) ensures that mixing between adjacent reagents is dominated by molecular diffusion (Atencia and Beebe 2005). This feature has been exploited to precisely control the spatial and temporal distribution of extracellular signals down to the single-cell level (Takayama et al. 2003; Wheeler et al. 2003). Recently, microfluidic probes (MFPs) have been used as non-contact tools for molecular gradient delivery on open substrates (Ali et al. 2026, 2025; Juncker et al. 2005). However, the long processing times often required for sequentially processing small areas and susceptibility to evaporation makes this technique unsuitable for sustained, long-term cell culture applications (Ali et al. 2026; Tourovskaia et al. 2005). Moreover, the use of MFPs becomes impractical when repeated measurements on the same cell populations are necessary (Qasaimeh et al. 2012).

Closed microfluidic systems have been used to generate complex biomolecular concentration gradients for studying cell proliferation, migration, and differentiation (Keenan and Folch 2007; Tehranirokh et al. 2013; Toh et al. 2014; Berthier and Beebe 2014). Diffusion-based gradient generators have leveraged geometric features (Cimetta et al. 2010; Bhattacharjee et al. 2010; Atencia et al. 2009 and porous materials (Auxillos et al. 2024; Samandari et al. 2021; Vandersarl et al. 2011) to deliver stable concentration gradients in low- or zero-shear environments. However, to accurately model how cells respond to biochemical gradients *in vivo*, it is necessary to replicate physiological and pathological shear stress conditions during delivery (Chen et al. 2019; De Stefano et al. 2022). Convection-dominated gradient generators have been used to produce complex, non-linear gradients with precise spatial control of parallel streams (Jeon et al. 2000; Dertinger et al. 2001; Holden et al. 2003; Cooksey et al. 2009; Zhou et al. 2009), enabling studies of cellular responses to concurrent gradients in shear stress and molecular concentration (Walker et al. 2005; van der Meer et al. 2010; Lin et al. 2019; Jaberi et al. 2020). However, these devices are typically irreversibly sealed, which limits their reusability and utility for biochemical assays that require cellular access (Mcdonald et al. 2000; Temiz et al. 2015).

Although several reversible sealing strategies have been integrated into convection-dominated gradient generators to deliver solute gradients under shear-controlled conditions to cells pre-cultured off-chip, their adoption has been limited by low throughput, insufficient sealing strength, and poor operational robustness (Teixeira Carvalho et al. 2023). Systems that use the self-adhesion of PDMS substrates (Wang et al. 2008) have burst pressures up to 22 kPa, and often require protocols that are incompatible with cell culture to achieve reportedly high burst pressures (Shiroma et al. 2016; Chu et al. 2017. Systems that feature vacuum sealing (Chung et al. 2007; Sip et al. 2011) have lower burst pressures (*<15* kPa) and require peripheral vacuum channels and a vacuum source that complicates device operability. Systems that integrate magnetic (Tkachenko et al. 2009) or mechanical clamping (Benedetto et al. 2014; Orcheston-Findlay et al. 2018; Auxillos et al. 2024 offer rapid and easy assembly to achieve high burst pressures (*<400* kPa) (Chen et al. 2014; Viefhues et al. 2017), but require custom holders to reliably seal PDMS microfluidic devices against cell-plated coverslips. Consequently, more robust clamping strategies are required to generate spatiotemporally controlled gradients and shear stresses for high-throughput assays involving post-treatment cell collection.

Here, we present a reversibly-sealable microfluidic platform that enables the simultaneous delivery of well-defined gradients of multiple small molecules with a wide range of shear stresses to adherent cells plated on centimeter-scale glass coverslips. Our device allows for the reversible integration of coverslips above microfluidic channels embedded in two irreversibly bonded layers of polydimethylsiloxane (PDMS), which are sealed during perfusion with a mechanical clamp. Our platform generates convection-dominated gradients from up to seven unique inputs with reliable sealing to *∼ 50* kPa and operates at Reynolds numbers ranging from the Stokes (*Re≪ 1*) to the laminar regime (*Re∼ 10*), enabling elevated flow conditions and a wider tunable range of cellular shear stresses (up to 1 Pa).

We experimentally and numerically evaluate the ability to spatially tune the mixing of each reagent stream in the chamber by altering the magnitude of the input flow rates. We show that gradients of multiple cell-permeable fluorophores can be precisely delivered to a confluent monolayer of fibroblasts across a chamber area of $\sim 4\text { cm}^2$. Cells remain viable after short-term (*∼ 30* minutes) perfusion and coverslip removal, which facilitates repeated experimentation and longitudinal treatment. These results highlight the feasibility of our microfluidic platform for large population cell-based assays under physiologically relevant conditions.

## Results

### Device design and fabrication

**Fig. 1:**
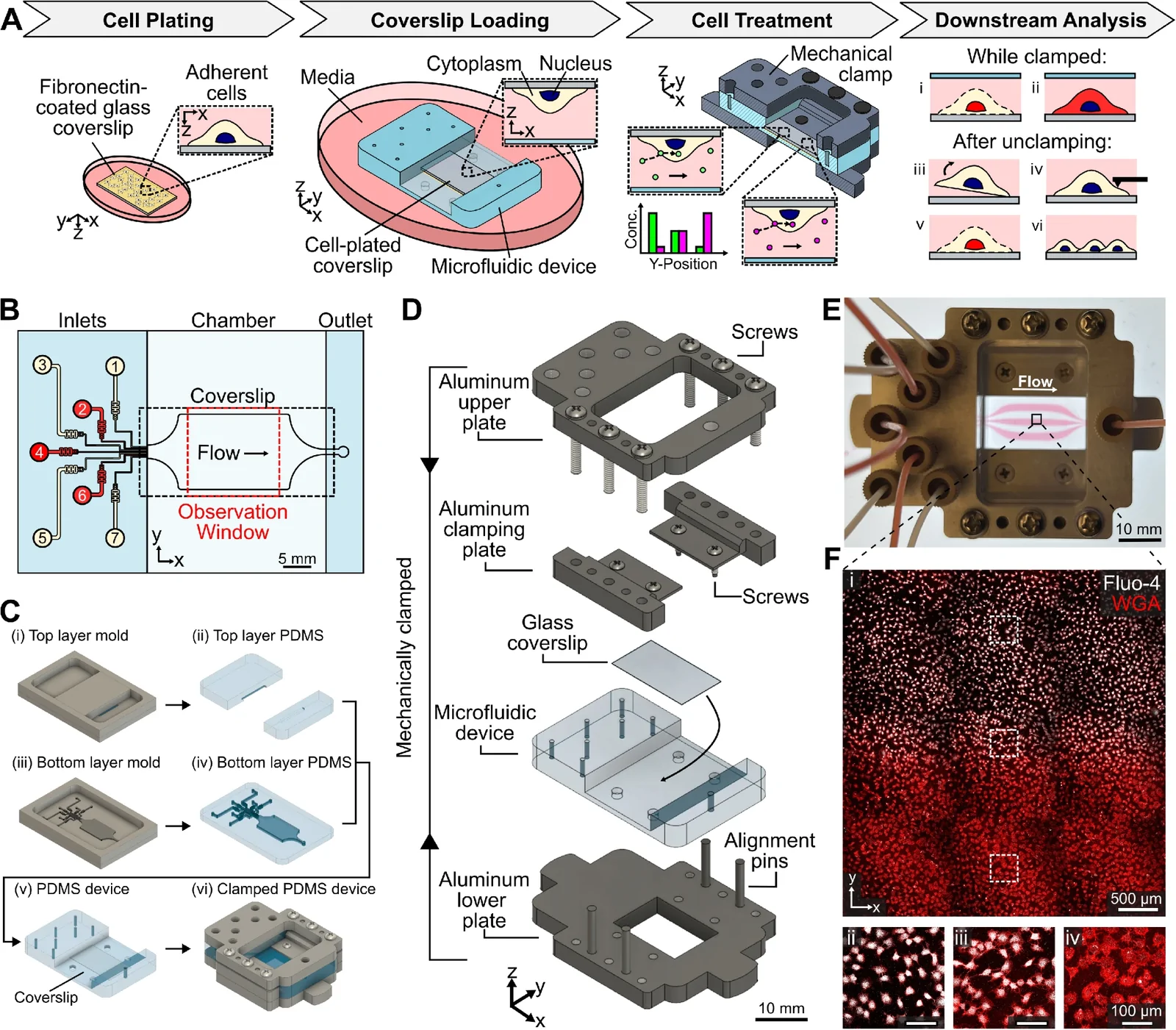
Microfluidic platform for high-throughput concentration-dependent studies on adherent cells. (**A**) Schematic workflow for exposing cells to concentration-gradients in the flow chamber. The procedure consists of: cell plating, coverslip loading, cell treatment, and downstream analysis. While the device is clamped, (i) live/dead staining and (ii) immunostaining can be performed. After unclamping the device, (iii) cell harvesting, (iv) mechanical testing, (v) immunostaining, and (vi) additional culturing can be performed. (**B**) Schematic of the microfluidic channel design of the reversibly sealable device. The network consists of seven inlets that connect to a fluidic chamber with one outlet. The observation window above the chamber is 20 mm long and 14 mm wide. (**C**) Schematic diagram of the reversibly-sealable, microfluidic device fabrication. (i-ii) The top layer PDMS molding process. (iii-iv) The bottom layer PDMS molding process. (v) The layers are plasma treated, aligned, and bonded using a custom built rig to form the PDMS device. (vi) The clamped PDMS device. (**D**) Schematic exploded isometric view of the multilayer mechanical clamp device for reversibly sealable, gradient flow generation. A glass coverslip is secured in the pockets of two irreversibly bonded PDMS fluidic layers, sandwiched between an aluminum housing, and clamped with a set of fasteners. (**E**) Photograph of a device with a laminar flow pattern generated in the chamber with red dye at a per-inlet flow rate of $25 \ \mu \text {L} \text { min}^{-1}$. (**F**) Magnified view of a tile scan acquired at the interface between adjacent streams of two different cell-permeable fluorophores. Cells exhibit concentration-dependent staining according to their position within each fluorophore stream. Panels (ii, iii, iv) show representative images along the lateral direction in the chamber (top, middle, bottom). Experimental conditions are described in 2.3 Selective multi-chemical delivery to adherent cells

We introduce a workflow to simultaneously screen responses to gradients of multiple small molecules on cells using reversibly-sealable microfluidic devices (Fig. 1A). First, adherent cells are plated on glass coverslips pre-treated with cell-attachment proteins (fibronectin or collagen). Cell-plated coverslips are loaded above a flow chamber in a microfluidic device. Subsequently, a metal chip holder connected to a perfusion system seals the coverslip in the device. Cells are treated with the desired quantities and types of reagents by continuously perfusing the chamber at specific flow rates. Finally, the coverslip can be removed from the device, which enables further downstream analysis, including immunofluorescence staining for morphology, viability, and molecular uptake.

The microfluidic platform is composed of a multilayer elastomeric microfluidic device coupled with a custom-made aluminum clamp. The microfluidic device is fabricated by irreversibly bonding two layers of polydimethylsiloxane (PDMS) to create ports for fluid injection and pockets to immobilize a glass coverslip (Fig. 1B, S1, S2). The lower layer of PDMS incorporates a series of delivery channels to deliver up to seven inputs to a large fluidic chamber that connects to a single outlet channel for solution collection (Fig. 1C). The upper layer of PDMS includes a space to fit microscope objectives for live cell imaging and analysis. The elasticity of the PDMS-based device allows for the repeated insertion and removal of glass coverslips.

To seal the microfluidic channels, a custom mechanical clamp consisting of a set of three aluminum plates fastened together with screws is used to sandwich the PDMS layers and coverslip (Fig. 1D); the clamp also maintains the mechanical stability of the device during handling. The upper plate includes threaded ports for the inputs and outputs and an observation window. The observation window is designed to accommodate objectives on an upright microscope with a free working distance down to 2.5 mm, enabling real-time live-cell imaging during treatment. The clamping plate is used to seal the edges of the coverslip against the lower layer of PDMS. The lower plate is used to secure the plates together and maintain the alignment of the device during imaging. Reagents are delivered to the device by a series of glass syringes connected to polytetrafluoroethylene (PTFE) tubing and polyether ether ketone (PEEK) fittings on a multi-rack syringe pump.

The mechanical sandwich clamp allows for perfusion up to a per-inlet flow rate of *700± 50* µL/min and a maximum burst pressure of 48 kPa before the laminar flow pattern fails and leaking is observed (Fig. S3). To ensure the shape of the gradient remains stable during perfusion, we employ on-chip bubble traps and submerge the device during assembly to minimize trapping air in the microchannels. Our devices can operate bubble-free continuously for at least 2 h without disruption to the gradient profiles.

Custom gridded glass coverslips are fabricated by photolithographic patterning and anisotropic etching (Fig. S4). Adherent cells are plated on the sterilized, gridded glass coverslips and sealed above the fluidic chamber in the microfluidic device. Perfusion of different concentrations and types of reagents through the device inputs enables combinatorial experiments with large numbers of cells. The dynamical features of the flow and concentration profiles are described by the Reynolds (*Re*) and Peclet (*Pe*) numbers. Here, $Re = U D_h/\nu$ and $Pe=\tau _D/\tau _C$, where *D* is the diffusion constant, $D_h$ is the chamber hydraulic diameter, *U* is the mean flow velocity, $\tau _D=W^2/D$ is the diffusive time scale (*W* is the chamber width), $\tau _C=L/U$ is the flow time scale, and *ν * is the kinematic viscosity. Importantly, our device can operate in a wide range of *Re*, from the Stokes (*Re≪ 1*) to the laminar (*Re∼ 10*) regime while keeping the high values of $Pe ~(>10^2)$. This allows for controlled concentration gradients and a wide range of imposed shear rates and stresses. Consequently, the amount of mixing between parallel streams allows for the selective treatment of cells with multiple small-molecule reagents (Fig. 1E and F). Due to the spatially resolved concentration gradients, cells receive position-dependent ratios of each reagent along the length of the coverslip at a desired shear stress. The etched grids allow specific regions of the coverslip to be mapped to reagent exposure conditions and facilitated relocation of the same cells for imaging following treatment.

### Flow and concentration gradient characterization

**Fig. 2:**
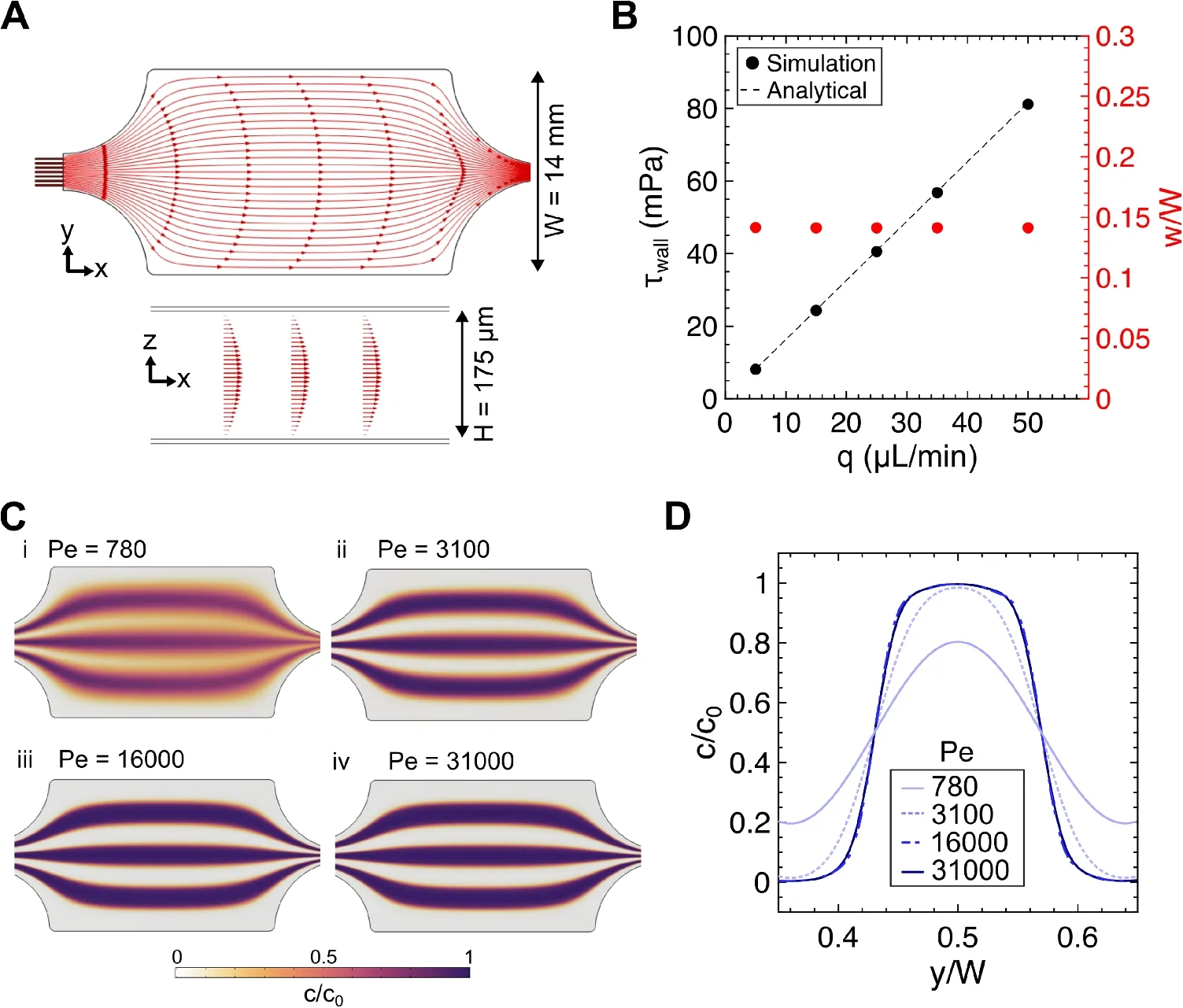
Numerical flow and transport simulations. (**A**) Top: streamline plot. Bottom: cross-sectional velocity profile; the length of the arrows is proportional to the magnitude of the velocity across the channel height. (**B**) Wall shear stress in the center of the chamber, $\tau _{wall}$, and peak width,$\bar{w}$, at half maximum height normalized by chamber width, *W*, as a function of per-inlet flow rate, *q*, at a constant diffusivity of $5\times 10^{-6} \text { cm}^2\text {s}^{-1}$. (**C**) Simulated concentration fields with Péclet number, $Pe = UW^2/LD$. The concentration is normalized by the initial concentration, $c_0$, at the inlets. (**D**) Simulation concentration profile of the center stream at the center of the chamber normalized by the max concentration as a function of the y-position normalized by the width of the chamber

To better understand the flow behavior within the central chamber of the microfluidic device, we perform finite-element numerical simulations using COMSOL. A 3D model of the chamber is generated with a height *H = 175* µm and center width $W = 14\ \text {mm}$; all flow simulations are performed at steady-state at comparable experimental *Re* and *Pe*. Upon entering the chamber, the flow diverges and aligns parallel to the channel walls before converging at the outlet (Fig. 2A). Along the chamber cross-section, the flow velocity shows the expected parabolic profile. The shear stress varies linearly along the walls, with the shear stress reaching a maximum in magnitude at the walls (Fig. 2B). The estimated wall shear stresses range from $8.2\pm 0.13 \text { mPa}$ to $82.2\pm 1.3 \text { mPa}$ for per-inlet flow rates of $5 \ \mu \text {L min}^{-1}$ to $50 \ \mu \text {L min}^{-1}$, respectively (Fig. S6). These values closely match the approximation of the maximum shear stress laminar flow between parallel-plates, $\tau _{max} = 6\mu \bar{Q}/WH^2$, where $\bar{Q}$ is the average total flow rate at the center of the chamber. Deen (2012) Per-inlet flow rates can be increased up to $600 \ \mu \text {L min}^{-1}$ to induce wall shear stresses up to $1 \text { Pa}$. Thus, our device can provide nearly 3 orders of magnitude of dynamical range in wall shear-stresses.

To gain further insights into the generation of tunable chemical gradients in the microfluidic device, we used finite-element numerical simulations to evaluate the transport of dilute species through the chamber. Briefly, for species with different diffusivities, we solved the advection–diffusion equation to model the combined effects of flow and molecular diffusion throughout the chamber (Fig. S5). The ratio of the convective transport to diffusive transport of reagents in the chamber is characterized by the Péclet number, $Pe = \tau _D/\tau _C$ (Deen 2012). The characteristic diffusive time scale is $\tau _D = W^2/D$, where *W* is the width of the chamber and *D* is the diffusivity of the species. The characteristic convective time scale is $\tau _C = L/U$, where *L* is the length of the chamber and *U* is the mean flow velocity at the center of the chamber. For typical molecules used in biological experiments (fluorescein, rhodamine, etc.), the diffusivity, *D*, ranges from $10^{-8}$ to $10^{-12}$ $\text { m}^2 \text { s}^{-1}$ (Toh et al. 2014). Therefore, the Péclet number in our microfluidic device at a per-inlet flow rate of $25 \ \mu \text {L min}^{-1}$ ranges from $7.8\times 10^2$ to $7.8\times 10^6$.

The steady-state concentration distribution in the chamber and the concentration profile of the central stream are measured for a range of Péclet numbers (Pe = 780-31000) (Fig. 2C and D). The concentration profiles show three distinct streams in the center of the chamber. At low Péclet numbers (Pe = 780), the solute experiences a competition between flow and diffusion that leads to a relatively broad band of concentration. By contrast, at high Péclet numbers (Pe *> 3100*), the concentration profile of each stream is quite sharp and step-like; this behavior is consistent with laminar flow at high Péclet numbers, where convection dominates transport in the fluidic chamber. These results show that concentration gradients can be tuned by varying the input flow rates, accommodating molecules with a range of diffusivities. At a constant diffusivity of $5\times 10^{-6} \text { cm}^2\text {s}^{-1}$, which is approximately the diffusivity of fluorescein in water (Jeon et al. 2000), and *Pe > 3100*, the width of each stream at half maximum peak height remains constant (Fig. 2B). To quantify the effective gradient steepness under varying flow conditions, we computed the secant slope between local maxima and adjacent minima in the concentration profiles as a function of the Péclet number, based on numerical simulation results. For *Pe=780*, the rate of change of the concentration was 0.31 units per mm. For *Pe>3,100*, the rate of change of the concentration was *0.50± 0.006* units per mm. The concentration profiles do change substantially at high Péclet numbers, as is expected since the system is dominated by convection and thus is diffusion limited. Therefore, we demonstrate that the flow rates can be modified to alter the magnitude of the wall shear stress without modifying the concentration profiles along the chamber axial direction, away from the chamber inlets and outlets. Indeed, one of the features of microfluidics is the ability to impose a wide range of shear stresses while keeping inertial forces negligible (low *Re*) (Pan and Arratia 2013). Together, these data show that one can perform shear stress dependent studies on adherent cells, while delivering a well-controlled concentration gradient across the chamber.

**Fig. 3:**
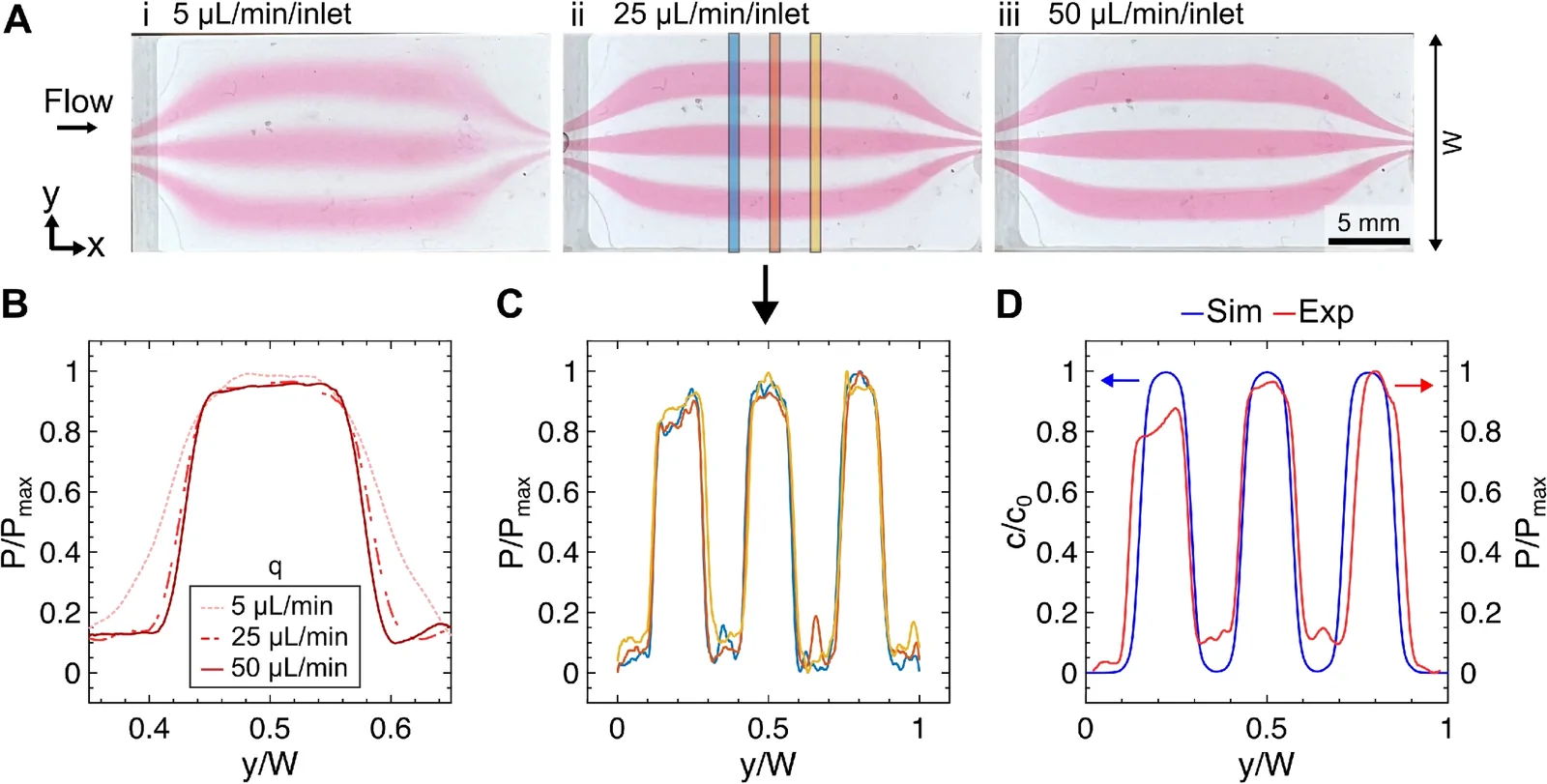
Experimental gradient characterization. (**A**) Experimental images of the chamber during gradient generation using red dye. (**B**) Experimental intensity profile, *P*, of the center stream at the center of the chamber normalized by the max intensity, $P_{max}$, as a function of the y-position normalized by the width of the chamber for various per-inlet flow rates, *q*. (**C**) Normalized intensity profile along the upstream, midstream, and downstream sections of the center of the chamber at a per-inlet flow rate of $25 \ \mu \text {L} \text { min}^{-1}$ as a function of the y-position normalized by the width of the chamber. (**D**) Steady state concentration profiles at the center of the chamber for red dye compared to the simulated profile at $25 \ \mu \text {L} \text { min}^{-1}$

Next, we compare the numerically obtained concentration gradients to microfluidic experiments by perfusing red ink and phosphate buffer through alternating inlets of the device. The device is operated at identical per-inlet volumetric flow rates, *Q*, of 5, 25, and 50 µL/min, as in the simulations; these values correspond to total flow rates, $\bar{Q}$, of 35, 175, and 350 µL/min (Fig. 3). The concentration gradient is characterized using colorimetric analysis based on spatial variations in dye intensity. Images of the chamber are captured using a CCD camera, and the steady-state grayscale intensity profiles in the axial and transverse directions in the chamber are processed to assess the amount of diffusion between the streams (Fig. 3A). Each image pixel value is normalized by the maximum pixel intensity value in each profile. In the transverse (y-) direction, the concentration profile of each stream transitions from a bell-shaped profile to a step profile as the flow rate increases (Fig. 3B). In the axial (x-) direction, we find a relatively stable concentration profile within the observation window of the chamber at $25 \ \mu \text {L} \text { min}^{-1}$, as shown by the profiles at three different locations along the axial direction in Fig. 3C). We note that at sufficiently high flow rates, the concentration gradient remains relatively constant (CV < 15%, p = 0.99) (Fig. S7) across the observation window in the flow chamber, which is characteristic for laminar flows at low *Re* and high *Pe* (Cooksey et al. 2009; Chang et al. 2014). The experimentally measured concentration profile exhibits non-uniform peak shapes, which can be attributed to variations in the local flow velocity across the channel and minor diffusion-induced broadening (Fig. 3D). Overall, we show that the device offers spatial control over diffusive mixing, which enables the delivery of combinations of different concentrations and chemical species in the chamber.

### Selective multi-chemical delivery to adherent cells

**Fig. 4:**
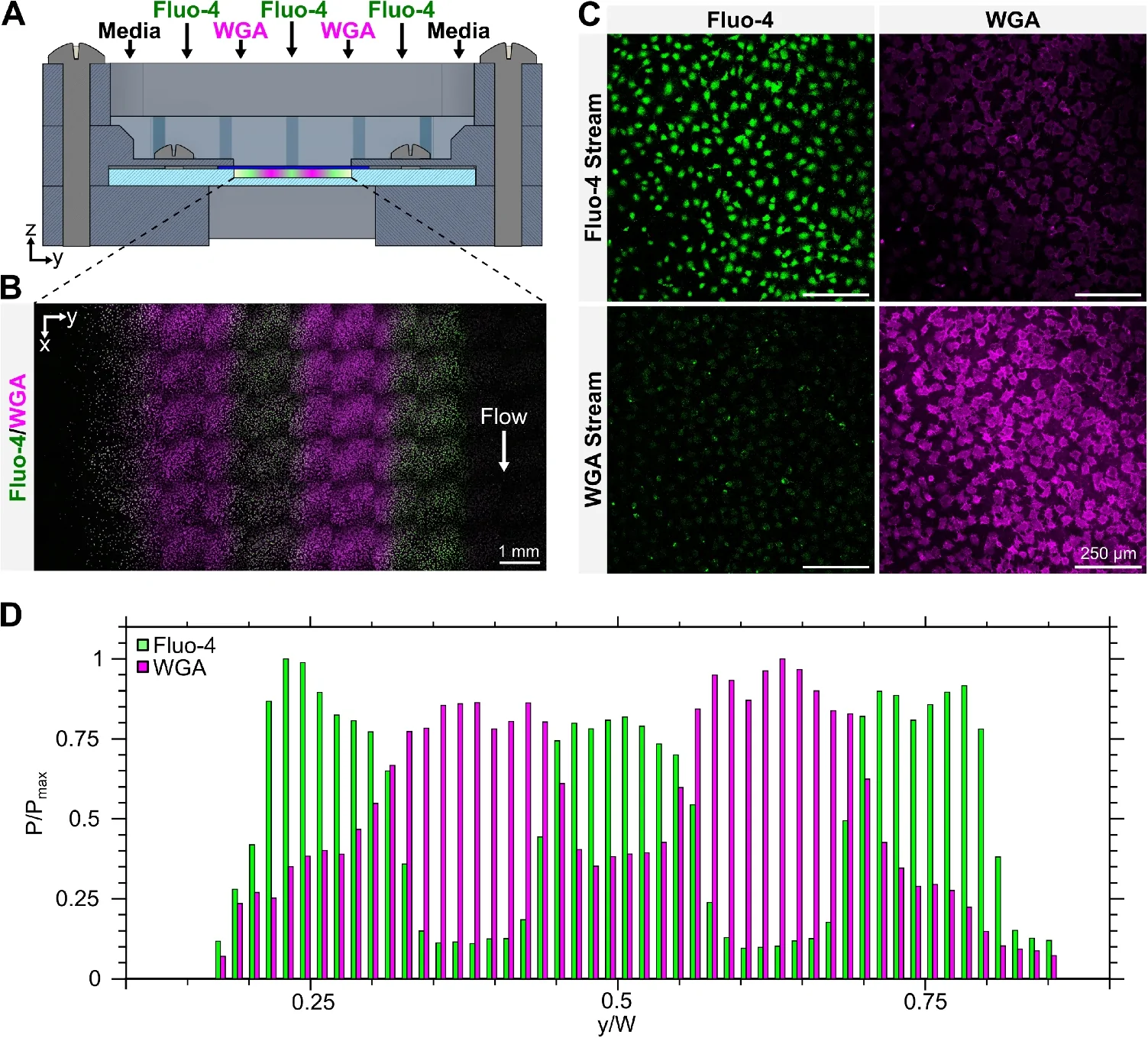
Multi-solute delivery to adhered cells. (**A**) Schematic of the cross-section of the reversibly-sealable microfluidic device for high-throughput treatment of adherent cells. (**B**) Fluorescence image of 3T3 cells stained with Fluo-4 and wheat germ agglutinin (WGA) after 30 min of perfusion at a per-inlet flow rate of 25 $\ \mu \text {L min}^{-1}$. (**C**) Representative fluorescence micrographs of concentration-dependent staining of 3T3 cells with Fluo-4 and WGA depending on their spatial position in the microfluidic chamber. (**D**) Fluorescence intensity of Fluo-4 and WGA normalized by the max fluorescence intensity in each stream averaged along the axial (x-) direction as a function of the y-position normalized by the width of the chamber

To demonstrate the feasibility of our microfluidic platform for reversibly sealing and selectively delivering molecules across cell-plated coverslips, we simultaneously expose cells to multiple cell-permeable fluorophore gradients. Adherent mouse 3T3 cells are plated on fibronectin-coated glass coverslips at a concentration of $3 \times 10^5$ cells/mL. As an indicator of cell confluency, we stain cellular DNA with Hoechst 33342. This coverslip containing the stained cells is sealed above the flow chamber of the microfluidic device. Next, a gradient of calcium ion indicator (Fluo-4) and glycocalyx stain conjugate (WGA, Wheat Germ Agglutinin) dissolved in cell media solution is established in flow (Fig. 4A). The diffusion coefficient of the dilute solutions of Fluo-4 and WGA used are $\sim 5\times 10^{-10} \text { m}^2 \text { s}^{-1}$ (Casalini et al. 2011). This value lies in the same range as the diffusivity of doxorubicin (Walsh et al. 2009) and certain growth factors (van der Meer et al. 2010), so it serves as a proxy for estimating the concentration profile for drug therapeutic and cell migration assays. After 30 min of perfusion, cellular uptake of the dyes is observed across the center of the coverslip (Fig. 4B). Diagonal intensity gradients observed within each tile are attributed to laser scanning artifacts during confocal tile acquisition. Cells that are located in the center of the Fluo-4 streams are stained green, while cells in the center of the WGA streams are stained pink (Fig. 4C). Cells that are located in the outer regions where only cell media is perfused are not stained by either fluorophore.

To quantify the ratio of fluorophore concentrations inside the cells, the fluorescence signal measured in each cell across the axial (x-) direction is averaged and discretized into 50 rows. The mean fluorescence intensity measured in the cells across the coverslip is proportional to the solution concentration gradient (Fig. 4D). Differences between the Fluo-4 and WGA profiles and the colorimetric profiles can be attributed to the lower detection limit inherent to fluorescence imaging. In addition, the concentration field is discretized, and variations in cell density across the coverslip, along with heterogeneous dye uptake dynamics, further contribute to deviations from the idealized flow-based behavior. These results demonstrate that gradients of multiple small molecules can be generated simultaneously across thousands of adherent cells for high-throughput functional assays.

### Cell viability after treatment and extraction

**Fig. 5:**
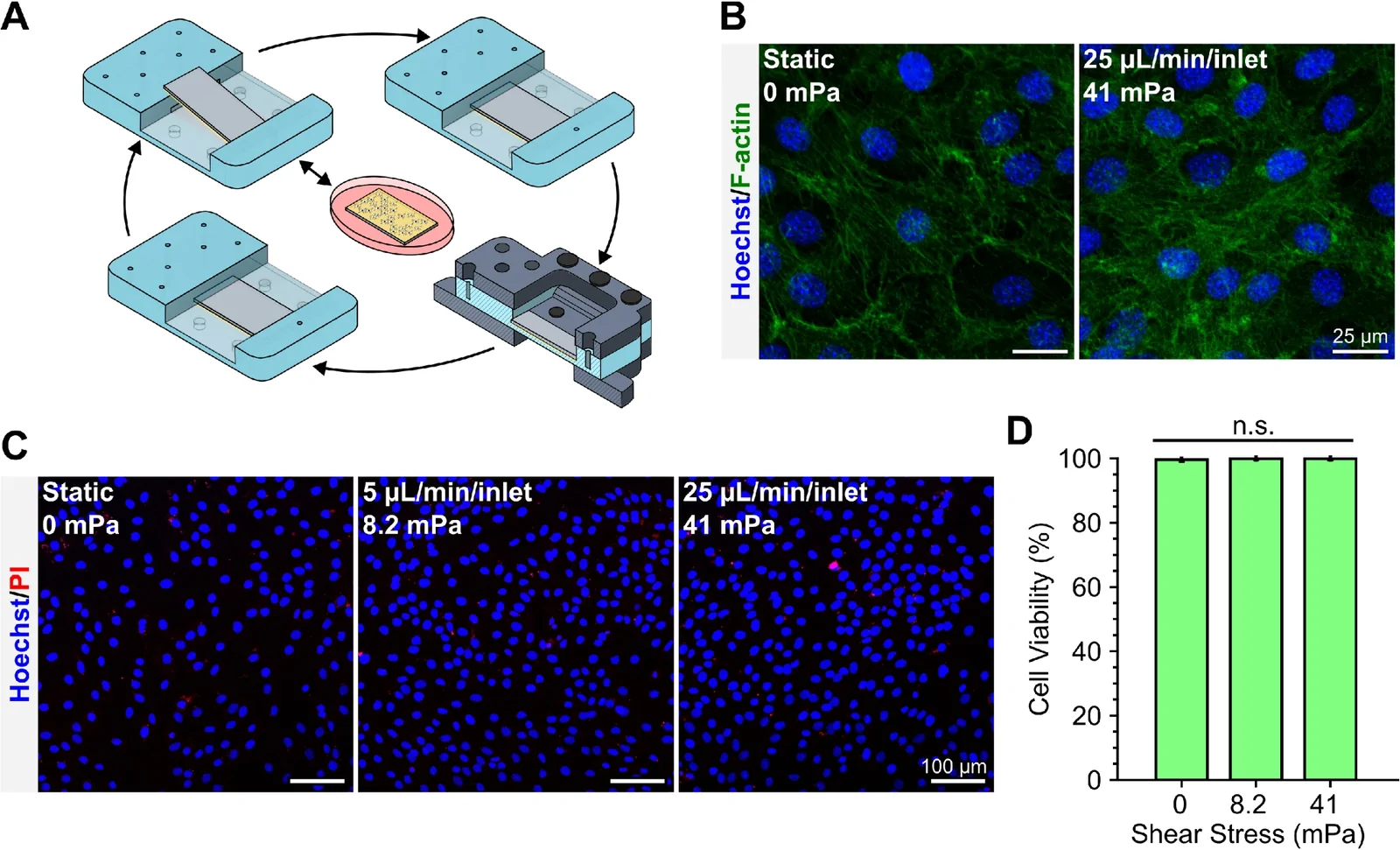
Cell viability after perfusion and removal from the reversibly-sealable microfluidic device. (**A**) Schematic workflow for coverslip integration and extraction. (**B**) Representative confocal fluorescence micrographs of cells stained for Hoechst 33342 (blue) and F-actin using phalloidin (green) after static conditions and exposure to flow-induced shear stresses of 41 mPa for 30 min. (**C**) Representative confocal fluorescence micrographs of cells stained for Hoechst 33342 (blue) and propidium iodide (PI) (red) after static conditions and exposure to flow-induced shear stresses of 8.2 and 41 mPa for 30 min. (**D**) No significant difference in cell viability between cells after 30 min of flow-induced shear compared to cells in static conditions (P > 0.05; n =3). Statistical significance determined by one-way ANOVA. Data shown as mean ± SEM. ns: Not significant

To determine the effect of coverslip handling and short-term flow-induced shear stresses on mammalian cells plated on the coverslips, we assess the morphology and viability of 3T3 cells after perfusion and removal from the device. We utilize the reversibility of the mechanical clamp and the compliance of the PDMS to gently insert and remove the coverslips into and out of the device for downstream analysis (Fig. 5A). Cell-plated coverslips are inserted into the device and perfused with cell media at per-inlet flow rates of 5 and 25 µL/min for 30 min. The 30 min viability assay was selected to evaluate acute cellular response during gradient establishment and perfusion, which represents the primary operational timescale investigated in this work. The minimum time for the gradient to stabilize across the length of the coverslip (30 mm) at this flow rate is about 3 min. The wall shear stress experienced by the cells in the center of the chamber is expected to be less than 100 mPa, which is not expected to affect 3T3 cell adhesion and viability (Truskey and Pirone 1990). After coverslip removal, the cells are fixed, permeabilized, and stained for F-actin to investigate the spreading and morphology of the cells. Cells retain normal spreading and morphology (Fig. 5B). No obvious changes in actin fiber alignment are observed at these total flow rates and treatment durations.

Cell viability is assessed by live/dead staining with Hoechst 33342 and propidium iodide (PI) (Fig. 5C). Cells exposed to 30% DMSO for 30 min in static conditions have a viability of 17% (Fig. S8). No significant difference in cell viability is observed between cells incubated in cell media under static conditions and cells exposed to flow-induced shear stresses. Cell viability remains *>99%* after short-term perfusion (30 min) and coverslip removal (Fig. 5D). The absence of detectable changes in cell morphology, confluency, or cell–cell junction integrity suggests that the platform does not measurably perturb the cellular state or bias cells toward specific responses following coverslip handling and low shear flow exposure, allowing downstream responses to be attributed primarily to the applied experimental stimuli. This demonstrates that this microfluidic device shows promise as a platform for functional assays requiring multiple treatments with intervening cell culture, additional “off-platform” manipulations such as biomarker assays (e.g. immunocytochemistry) or single-cell harvesting.

## Methods

### Device design and fabrication

The lower PDMS layer was fabricated using photolithography and soft lithography techniques. The channels were designed in LayoutEditor (juspertor GmbH) and patterned onto chrome-coated soda lime photomasks (AZ1500) using a laser writer (Heidelberg 66 DWL+) with a 10 mm write head. Photomasks were developed in AZ 300MIF developer, etched with chromium etchant, and any remaining photoresist was stripped with NMP/KL Remover. To fabricate the master mold for the lower PDMS layer, SU-8 2050 (MicroChem) was spin-coated to a thickness of 175 µm on a 500 µm thick, 100 mm silicon wafer (University Wafer, 452). The channels were lithographically patterned onto the photoresist using a mask aligner (SUSS MicroTec) followed by development in SU-8 developer (MicroChem). The master was rendered hydrophobic by vapor deposition of (1 H,1 H,2 H,2 H-perfluorooctyl)-trichlorosilane (Sigma-Aldrich, 448931).

A set of aluminum assemblies were machined to ensure consistent PDMS layer dimensions during casting and layer alignment during bonding. For the lower PDMS layer, the silicon master was sandwiched between a 1/8" aluminum plate machined with a rectangular gap and a 1/4" aluminum base plate. The cavity formed by the rectangular gap was centered above the extruded photoresist channels. For the upper PDMS layer, a custom glass coverslip was sandwiched between a rectangular aluminum block inserted in the gap in the 1/4" aluminum plate and a 1/4" aluminum base plate to form two cavities. The edge of a 200 µm thick, 32 mm x 18 mm rectangular glass coverslip protruded by 1 mm on either side of the rectangular block. The cavities in the lower and upper layer assemblies were filled with a degassed 10:1 mixture of polydimethylsiloxane (PDMS, Dow Sylgard, 184). PDMS casting was performed as previously described by Cameron et al. (2022), with minor modifications. To maintain the thickness uniformity of the PDMS layers, a transparency film (Apollo, VCG7070), glass slide, and a weight were layered above the PDMS-filled cavities before curing the casting assemblies in an oven at 70$^{\circ }$C for at least 3 h.

A bonding guide was machined out of 1/4" aluminum plate with the same gap dimensions as the lower PDMS layer casting plate. The cured PDMS slab for the lower layer was placed channel-side up in the rectangular gap. The cured PDMS slabs for the upper layer were placed pocket-side down on the respective inlet and outlet sides of the lower PDMS layer slab. A 1.5 mm biopsy punch (Thermo Fisher) was used to punch holes for the inlets and outlet in the upper PDMS layer slabs. The lower and upper PDMS layer slabs were exposed to oxygen plasma and inserted into the bonding assembly in the same order as stated before to irreversibly bond the layers together. The bonded device was heated on a hotplate at 80$^{\circ }$C for at least 1 h to ensure complete bonding. The final thickness of the upper and lower PDMS layers is 1/4" and 1/8", respectively.

### Experimental assembly and operation

To clean the devices before each use, the PDMS devices were immersed and sonicated in 70% ethanol for 10 min, degassed in DI water for 10 min, and dried in air at room temperature. A syringe containing DI water or serum-free media was connected to the device inlets and outlet via needle and tubing, and the device was primed to remove any trapped air bubbles.

To insert the glass coverslips without introducing air into the chamber, the device was loaded onto a custom uniaxial stretcher and immersed in a DI water or serum-free media bath. The inlet-side edge of a coverslip was aligned with the inlet-side pocket of the device. The device was stretched by rotating the screw on the stretcher until the coverslip sat flush with the chamber surface. After the coverslip was aligned with the pockets, the device was relaxed to its unstrained configuration.

To seal the fluidic chamber in the microfluidic device to the glass coverslip, a custom mechanical clamp was designed in Fusion 360 (AutoDesk) and machined out of 6.35 mm thick 6061 aluminum. The upper plate featured eight 1/4-28" UNF threaded ports aligned above the ports for the inlets and outlet and a window for an upright microscope objective to translate above the microfluidic chamber. The clamping plate included four 1/4-20" UNF through holes for fasteners to seal the outer edges of the glass coverslip to the lower PDMS layer. The thickness of the clamping plate along the area that interfaces with the coverslip are designed to accommodate objectives with a working distance down to 2.5 mm. The lower plate included a window to allow full illumination of the coverslip. All plates featured four 1/8" diameter holes with slip-fit tolerance to accommodate 1/8" diameter, 3/4" length dowel pins for aligning the plates and six through holes for fasteners to secure the assembly together.

The gaps in the 0.25" thick upper and lower clamping plates were designed to allow for imaging live-cells in the device on both upright and inverted microscopes. The dimensions of the upper clamping plate gap is 44 mm by 29 mm, which accommodates objectives on an upright microscope with a working distance down to 2.5 mm and diameters up to 27.5 mm. This allows an objective to translate along the y-direction of the chamber to image cells along the width of the chamber. The dimensions of the lower clamping plate gap are 28 mm x 20 mm, which accommodates objectives on an inverted microscope with working distance exceeding 6.35 mm.

To assemble the mechanical clamp, the device was placed on the aluminum lower plate. The aluminum clamping plates were fitted in the gap between the slabs of the upper PDMS layer. The aluminum upper plate was aligned above the clamping plates, and all the plates were fastened together via six 6-32 pan head screws positioned along the exterior of the plates. Four 2-56 flat head screws were inserted in the interior of the aluminum clamping plates and tightened to the aluminum lower plate until resistance was felt.

The fasteners on the clamping plate and upper plate were tightened to 5 cN*· *m and 10 cN*· *m, respectively, using a torque screwdriver (GearWrench 89625, 1/8" drive). We use the short form torque-preload equation, *T=FKD*, to calculate the clamping force, *F*, at the applied torque, *T*, assuming a nut factor, *K*, of 0.2, which is appropriate for dry stainless steel screws (Bickford 2008). This corresponds to a clamping force of approximately 140 N per 6-32 pan head screw on the upper plate and approximately 100 N per 2-56 flat head screw on the clamping plate. If a torque exceeding 10 cN*· *m is applied to the fasteners on the clamping plate, the coverslip may crack (Fig. S3).

Seven 1 mL glass syringes (Hamilton, 1001TLL) were filled with defined solutions, and connected to 20 gauge blunt tip needles (McMaster, 75165A677) and 1/16" OD PTFE tubing (Fluorostore, F015091). Flangeless fittings (IDEX, XP-283) were secured to the end of the tubing, with 2 mm of tubing sticking out of the ferrules. Syringes were loaded on a syringe pump with a multi-syringe rack (Harvard Apparatus PHD 2000). The tubing was primed with the solutions to prevent air bubbles from entering the device and inserted into inlets of the device. Solutions were injected into the microfluidic device at defined flow rates, where total flow rate refers to the flow rate through each inlet multiplied by the number of inlets (7). An 8" long PTFE tube with a flangeless fitting was connected to a 1 mL syringe, primed with DI water, and inserted into the outlet of the device. The syringe was lowered 2" below the level of the device and disconnected from the tubing to initiate gravity-driven flow. The gravity-driven flow at the outlet is utilized to minimize "end effects," such as bubbles, surface tension issues, and adverse pressure gradients, that may disturb the laminar flow pattern in the chamber.

After flow experiments, the clamp pieces are dried and the holes are cleaned with Simple Green Original (ULINE, S-20941) using pipe cleaners to remove any oxidation.

### Computational fluid dynamics simulations

COMSOL Multiphysics Version 6.1 (COMSOL, Inc. Burlington, MA) was used to evaluate shear stress, flow profiles, and particle transport in the chamber (Fig. S4). The 2D channel geometry was imported and extruded to a height of 175 µm to create the 3D model. The ’Creeping Flow’ module was used to run a stationary solver governed by the incompressible, Navier–Stokes equations to compute the velocity and pressure fields for the flow of water. All 7 inlets were prescribed with a fully-developed flow with a volumetric flow rate, *q*. The outlet was prescribed with a static pressure boundary condition of 0 Pa. All walls were prescribed a no-slip boundary condition. The ’Transport of Diluted Species’ module was used to run a stationary solver governed by the convection-diffusion equation to calculate the concentration field of a dilute solute in water. The velocity field solved in the ’Creeping Flow’ module was used to evaluate convective transport. No flux and an initial concentration of 0 was set in the domain. The concentration at inlets 2, 4, and 6 was set to $c_0 = 5\ \text {mol m}^{-3}$, and the concentration at inlets 1, 3, 5, and 7 was set to $0\ \text {mol m}^{-3}$. The diffusion coefficient, *D*, of the species was user-defined. A user-controlled, unstructured mesh was created with custom element size parameters calibrated for fluid dynamics with a maximum element size of 128 µm. The simulation results were exported and analyzed using a custom MATLAB (MathWorks) script.

### Experimental flow characterization

Red ink (Pelikan 4001) was diluted in Dulbecco’s Phosphate Buffered Saline (DPBS, Gibco) and used for visualizing gradient generation and stability in the microfluidic chamber. Each of the seven inlets was perfused with solution at the same flow rate to yield the designated total flow rates. After the steady state pattern was reached, photographs were taken of the device and the microfluidic chamber. The RGB-image was imported into a custom MATLAB (MathWorks) script and converted into grayscale. The background was subtracted from each intensity profile and normalized to the maximum intensity.

To assess the sealing reliability of the mechanical clamp, the maximum flow rate and maximum pressure sustaining leak-free operation was measured across multiple devices. The maximum flow rate was determined by perfusing all of the inlets of the device with aqueous solutions at the same flow rates using a syringe pump until leaking was observed. A burst pressure test was performed to determine the maximum pressure following the protocol described by Rafat et al. (2009). Briefly, a nitrogen tank was connected to a dual-valve pressure controller (Alicat Scientific, Tucson, AZ), which was in turn connected to a water reservoir containing red dye. The reservoir was connected to inlet 3 of the clamped device via 1/16" OD PTFE tubing, while all other inlets were sealed. The pressure was gradually increased until the dyed solution could be seen leaking out of the device.

### Coverslip preparation

Custom gridded glass coverslips were fabricated as cell culture substrates. To enable repeated cell localization after treatment, glass coverslips were patterned with a permanent reference grid using standard photolithography and reactive ion etching (RIE). Briefly, 200 µm-thick, 100 mm Borofloat 33 glass wafers (University Wafer, 2248) were spin-coated with a S1813, soft-baked, and exposed through a transparency film photomask containing eight duplicate grid patterns to enable parallel fabrication. Each pattern was set inside a 32 x 13.2 mm rectangle divided into 500 x 500 µm subdivisions, with a central horizontal axis numbered 1–59 and a central vertical axis lettered A–Y for spatial referencing. Following development, the exposed glass was etched using fluorine-based RIE, producing etched lines with a depth of approximately 100 nm. The etched wafer was subsequently cleaned in KL remover for 10 min, then NanoStrip for 10 min, followed by acetone, isopropyl alcohol (IPA) and deionized water for 5 min. The wafers were diced into 32 mm x 18 mm rectangular coverslips using a dicing saw (ADT 7122). Alternatively, commercially available gridded square coverslips could be used, but the desired coverslip would need to be used in the mold for casting the upper layer of PDMS. Glass coverslips were exposed to UV light in a sterile Petri dish for 30 min. Subsequently, each coverslip was coated with 500 µL of 0.1 mg/mL fibronectin or collagen. After incubating overnight at 37$^{\circ }$C, the coverslips were washed three times with sterile PBS. Prior to cell seeding, the washed coverslips were UV-treated for an additional 30 min.

### Cell preparation

NIH 3T3 fibroblasts (ATCC) were cultured in DMEM with 10% FBS (Sigma-Aldrich), 0.1 µg/mL penicillin, and 0.1 mg/mL streptomycin (Mediatech) in standard conditions (37$^{\circ }$C, 5% $\text {CO}_2$). Cells were detached using trypsin-EDTA (Sigma-Aldrich), neutralized with medium, and collected by centrifugation. The pellet was resuspended and counted using a Trypan Blue stain to determine initial viability (*>95*%). A suspension of $3 \times 10^5$ cells/mL was prepared, and 150,000 cells per coverslip were seeded by adding 500 µL of the suspension directly onto each collagen-coated coverslip. Cell-plated coverslips were incubated overnight to allow for cell adherence and spreading.

### Molecular gradient delivery

3T3 cell-plated coverslips were washed once with sterile PBS. 500 µL of culture medium containing 5 µg/mL Hoechst 33342 (Invitrogen, Thermo Fisher, R37165) was added to the coverslips and incubated for 30 min at 37$^{\circ }$C (in the dark). Fluo-4, AM (Invitrogen, Thermo Fisher, F14201) was diluted in serum-free media to yield a final concentration of 5 µM. Wheat Germ Agglutinin (WGA) conjugated to Alexa Fluor 647 (Invitrogen, Thermo Fisher, W32466) was diluted in serum-free media to yield a final concentration of 20 ug/mL. Microfluidic devices were fully immersed in a serum-free media bath, and the inlets and outlet were flushed with media to clear any trapped air bubbles. Coverslips were removed from their Petri dishes and placed with the cell-side facing the channels in the device. The device was perfused with 3 inputs of Fluo-4 solution (inlets 2, 4, 6), 2 inputs of serum-free cell media (inlets 1 and 7), and 2 inputs of WGA solution with blue food coloring (inlets 3 and 5) for 30 min. All seven inlets were operated at the same volumetric flow rate of 25 µL/min, yielding a total flow rate of 175 µL/min. After perfusion, the coverslip was removed from the device, washed with sterile PBS, and immediately imaged with an inverted confocal microscope (Leica DMi8) with a 10X objective. Fluorescent tile scan images were analyzed using ImageJ (NIH). Each fluorescent channel was thresholded to identify the cells, and cell intensities were extracted for analysis on a custom MATLAB (MathWorks) script. The average intensity profile for each fluorophore was normalized to the maximum intensity in each corresponding fluorophore stream.

### Cell viability

3T3 cells (50,000 per sample) were seeded onto collagen-functionalized glass coverslips prepared as described previously and cultured for 48 h. Cells plated on glass coverslips were incubated under static conditions for 30 min either in serum-free medium alone or in serum-free medium containing 30% DMSO. In parallel, additional samples were perfused with pure serum-free medium at per-inlet flow rates of 5 and 25 µL/min for 30 min. Following treatment, cells were incubated with propidium iodide (PI, 1 µg/mL; Invitrogen, Cat. No. 3566) in PBS for 30 min, fixed with 3.7% paraformaldehyde for 10 min at room temperature, washed with PBS, and permeabilized with 0.1% Triton X-100 for 10 min. F-actin was stained using AlexaFluor 488–conjugated phalloidin (Invitrogen, Cat. No. R37110) according to the manufacturer’s instructions, followed by nuclear counterstaining with Hoechst 33342 (5 µg/mL; Invitrogen, Thermo Fisher, H1399) for 30 min at room temperature. Coverslips were washed with PBS and mounted with an anti-fade reagent (Invitrogen, Cat. No. P36982) prior to confocal microscopy. Cells were imaged with an inverted confocal microscope (Leica DMi8) with a 20X objective. Fluorescence images were imported into ImageJ and at least 200 cells per condition were automatically detected. Viability was measured by calculating the ratio of cells negative for PI and positive for Hoechst (live cells) to cells positive for Hoechst (total cells).

## Discussion

We present an approach to deliver well-defined chemical concentration gradients of small molecules to adherent cells with tunable shear stresses. Our device achieves stable gradient generation in less than 5 min over cell culture areas of $\sim 4\text { cm}^2$. By incorporating a mechanical clamp to reversibly seal cell-plated glass coverslips above a flow chamber, we can perform repeated combinatorial experiments on the same population of cells for long-term, longitudinal biological studies.

Our device provides several practical advances over previously reported mechanically clamped, reversibly sealed gradient generators (Table S1) (Auxillos et al. 2024; Orcheston-Findlay et al. 2018; Benedetto et al. 2014). Notably, the cell culture chamber area ($\sim 4 \text { cm}^2$) exceeds that of prior systems, enabling treatment of substantially larger cell populations in a single experiment. At the cell density used here ($350 \text { cells/mm}^2$), this corresponds to the simultaneous exposure of approximately 147,000 cells, improving experimental throughput and accelerating the identification and validation of functional biological responses. In addition, the mechanically clamped architecture enables reliable sealing of pre-plated coverslips at pressures up to 48 kPa, expanding the operational robustness of the platform. In comparison, vacuum sealed microfluidic devices for convection-dominated gradient generation can typically operate up to pressures in the range of 0.1-15 kPa (Chung et al. 2007; Sip et al. 2011). The combined increases in chamber area and burst pressure broaden the accessible flow conditions, allowing operation across Reynolds numbers ranging from 0.01 to 10. This extended range permits application of wall shear stresses spanning both Stokes-dominated and higher laminar flow regimes, whereas most microfluidic gradient generators operate primarily within the Stokes regime. Although prior mechanically clamped microfluidic devices have allowed leakage (Benedetto et al. 2014) or utilized hydrogel barriers (Auxillos et al. 2024) to mitigate shear stresses, these approaches inherently limit precise and reproducible control over the physiological shear conditions experienced by cells. Collectively, these features expand the operational dynamic range of the device, with improvements arising from three primary factors: increased culture area, enhanced sealing strength, and an enlarged accessible hydrodynamic regime.

By incorporating reversible sealing, the platform allows for reuse, reducing fabrication costs and supporting repeated gradient-generation experiments within a single device. Because the device is fabricated from PDMS, potential absorption of certain organic solvents and hydrophobic molecules should be considered, particularly during repeated use (Toepke and Beebe 2006; Mukhopadhyay 2007). Notably, compounds with partition coefficients (*łog P < 2.47*) are expected to exhibit minimal absorption (*<10*%) into PDMS channels following 30 min of incubation at 25 $^{\circ }$C (Wang et al. 2012). To reverse any potential contamination, our devices are cleaned between experiments and the channels are rinsed with media before chemical treatment (Toepke and Beebe 2006). Various strategies for modifying the PDMS channels exist that can reduce the absorption of hydrophobic molecules (Cherukuri et al. 2026; Zhou et al. 2012). Our devices can be reused more than 20 times over a period of 6 months without degradation.

We ultimately envision that these devices could be integrated with automated, high-control intracellular delivery techniques, such as light-induced phototransfection, to address limitations to screening throughput (Brooks et al. 2020). Transcriptome-Induced Phenotype Remodeling (TIPeR) has been utilized for reprogramming cells via intracellular delivery of RNA populations (Kim et al. 2024; Cappelleri et al. 2010; Sul et al. 2009; Barrett et al. 2006). However, as published, only a single concentration of RNA was administered to the cells during phototransfection. To provide robust analysis of relative mRNA populations in cells, multiple concentrations of RNAs need to be administered in a defined robust manner. The ability to deliver multiple molecules at different ratios by controlling the fluidic inputs and flow rates will allow for rapid, physiologically-relevant screens across different RNA populations.

By generating predictable chemical gradients with cellular spatial resolution across centimeter-scale areas, this approach makes it possible to interrogate how heterogeneous microenvironments drive differential cellular responses within the same population, revealing emergent behaviors, threshold effects, and spatial dependencies that are otherwise obscured. Biologically, this capability is essential for linking molecular signaling dynamics to functional outcomes in complex cellular systems and for improving the physiological relevance and predictive power of *in vitro* models used to study disease mechanisms and therapeutic responses.

## Conclusion

In summary, we present a reversibly-sealable microfluidic platform that can be used to spatially control the simultaneous delivery of well-defined chemical concentration gradients of small molecules to centimeter-scale areas of adherent cells at low shear stresses. Our devices can be used for screening adherent cells that are not suitable for seeding directly in a microfluidic chamber, including primary neurons. The mechanical clamp enables leak-free perfusion at dynamically tunable shear stresses for replicating physiologically relevant conditions during molecular delivery. We believe that the platform demonstrated here provides a potential means of improving the throughput and clinical relevancy of high-control transfection methods.

## Supplementary Information

Below is the link to the electronic supplementary material.

Supplementary file 1 (PDF 22.5 MB)

## Data Availability

All data supporting the findings of this study are available within the paper and its Supplementary Information.
