# Supplementary material for: Reversibly-sealable microfluidic platform for multi-molecule gradient delivery to large adherent cell cultures
Source: Biomed Microdevices. 2026 Jun 17;28(3):50. doi: 10.1007/s10544-026-00831-z (PMC13275633; doi:10.1007/s10544-026-00831-z)
Supplement: Supplementary file 1 — (PDF 22.5 MB) [file 10544_2026_831_MOESM1_ESM.pdf]

Supplemental Information

Reversibly-sealable microfluidic platform for multi-molecule gradient delivery to large adherent cell cultures

Julia Radzio[1], Łukasz Suprewicz[2], Da Kuang[3], Alexander Karpowicz[1], Paul A. Janmey[2], Jai-Yoon Sul[3], David A. Issadore[4], James H. Eberwine[3], and Paulo E. Arratia[1]

[1]Department of Mechanical Engineering and Applied Mechanics, School of Engineering and Applied Science, University of Pennsylvania, Philadelphia, PA, USA
[2]Department of Physiology and Institute for Medicine and Engineering, Perelman School of Medicine, University of Pennsylvania, Philadelphia, PA, USA
[3]Department of Systems Pharmacology and Translational Therapeutics, Perelman School of Medicine, University of Pennsylvania, Philadelphia, PA, USA
[4]Department of Bioengineering, School of Engineering and Applied Science, University of Pennsylvania, Philadelphia, PA, USA

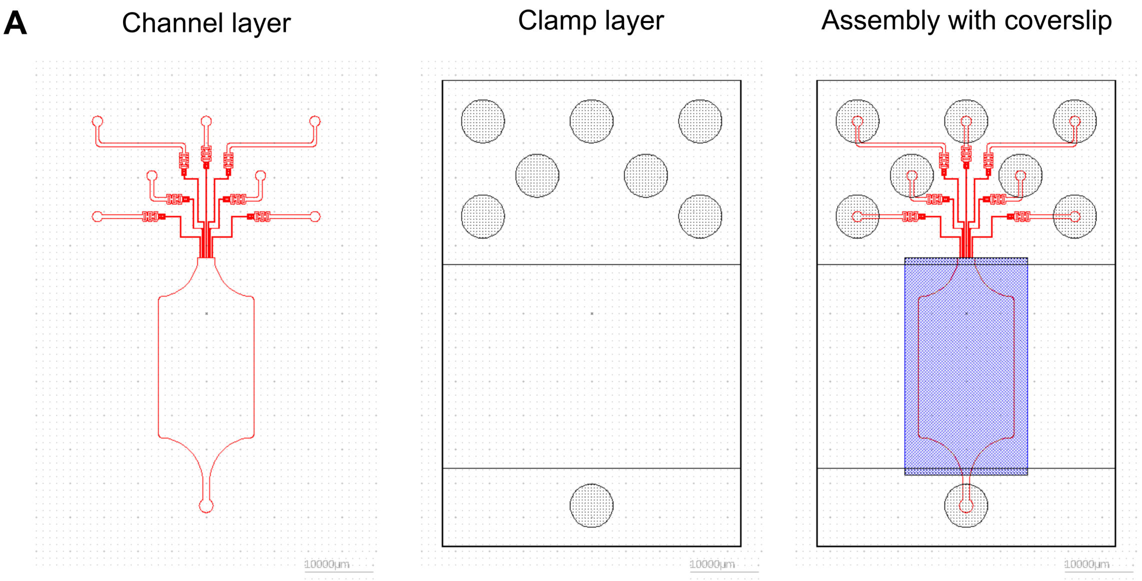

Figure S1: Microfluidic device design. A) Schematic of the top view of the microfluidic channels, mechanical clamp, and full assembly with the coverslip.

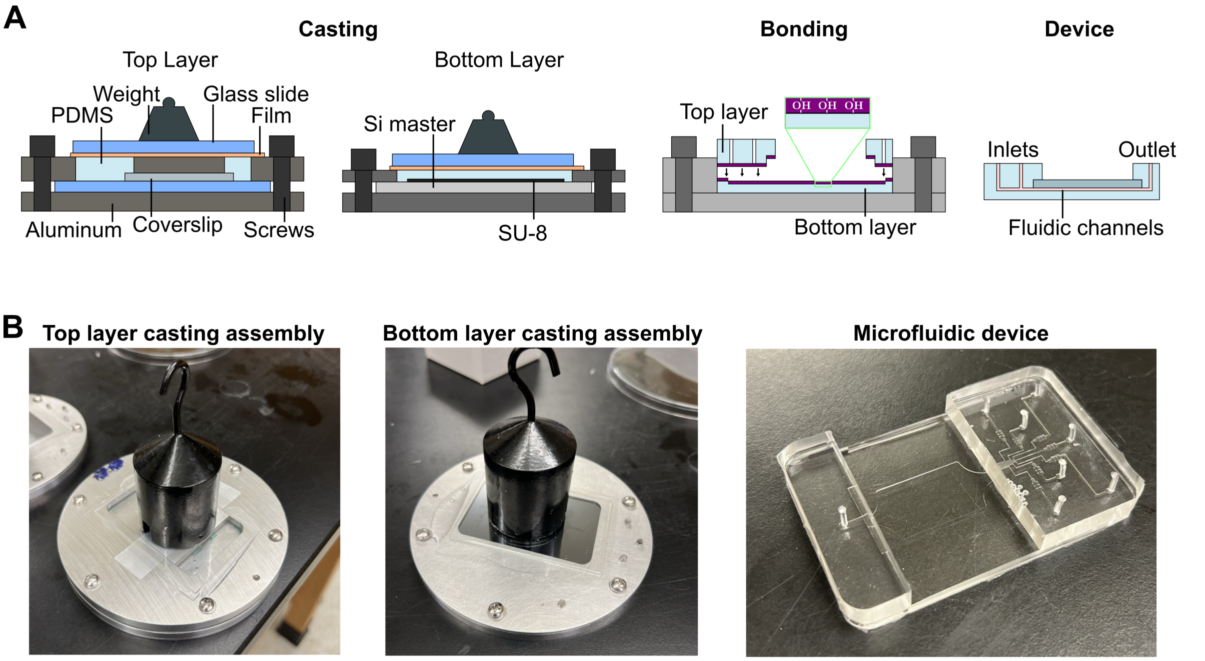

Figure S2: Molding process for the multi-layer microfluidic device. A) Schematic procedure for casting each layer, bonding the layers after plasma treatment, and the final microfluidic device. B) Images of the top layer casting assembly, bottom layer casting assembly, and bonded microfluidic device.

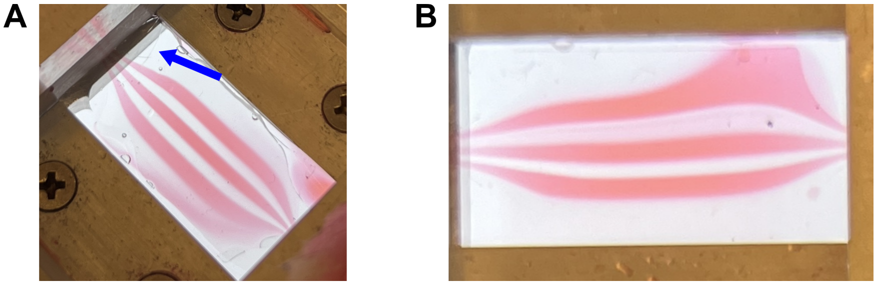

Figure S3: Seal failure. A) A torque exceeding 10 cN·m applied to the fasteners on the clamping plate can cause the coverslip to crack. The blue arrow indicates the location of the crack. B) Leakage occurs at the interface between the clamping plate and the microfluidic device at a per-inlet flow rate of 700 µL/min.

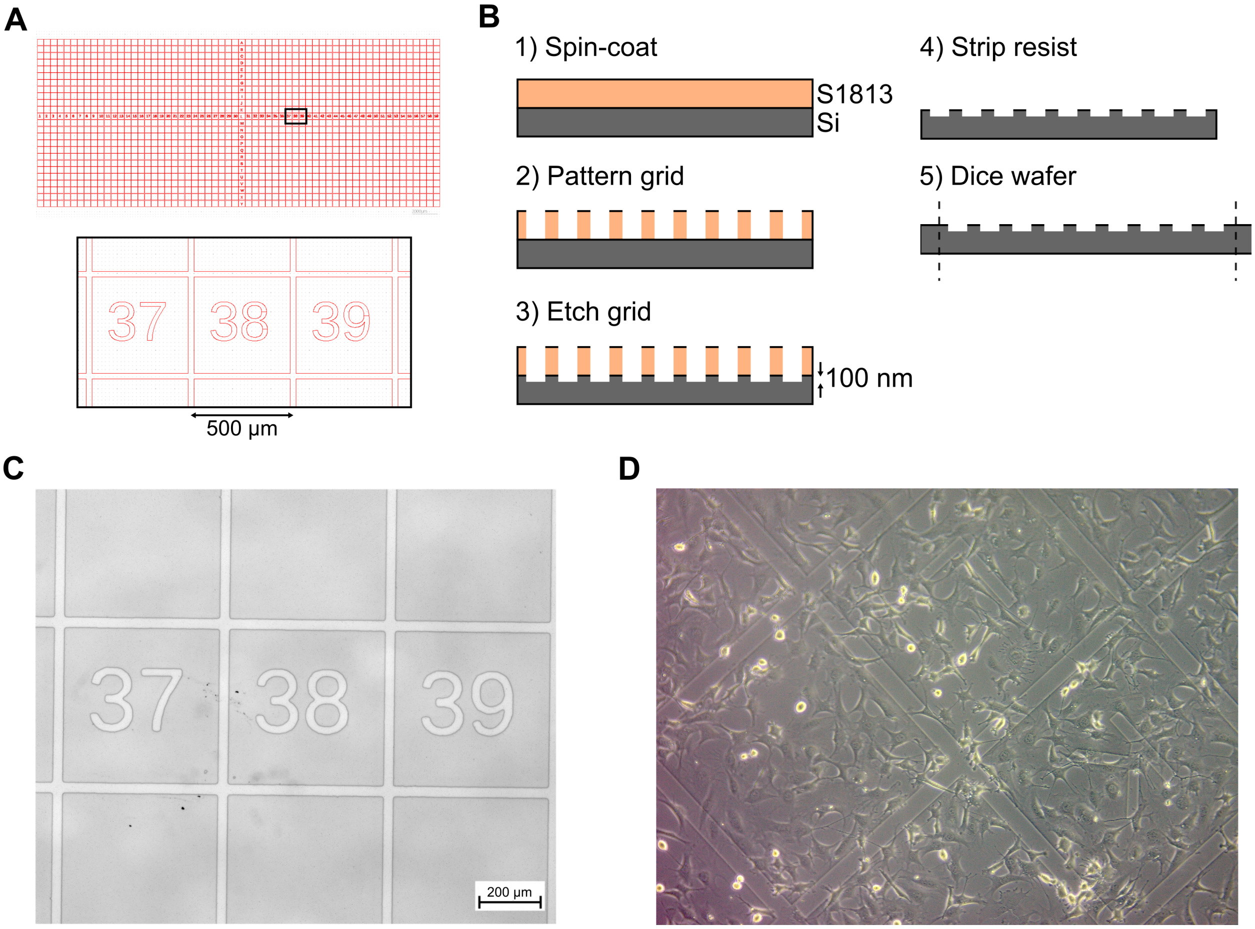

Figure S4: Custom glass coverslip fabrication protocol. A) Layout of the pattern on the gridded glass coverslips. B) Schematic step by step description of glass coverslip grid patterning, etching, and dicing. C) Representative image of a glass coverlip after fabrication. D) Representative image of PC12 cells plated on a gridded glass coverslip.

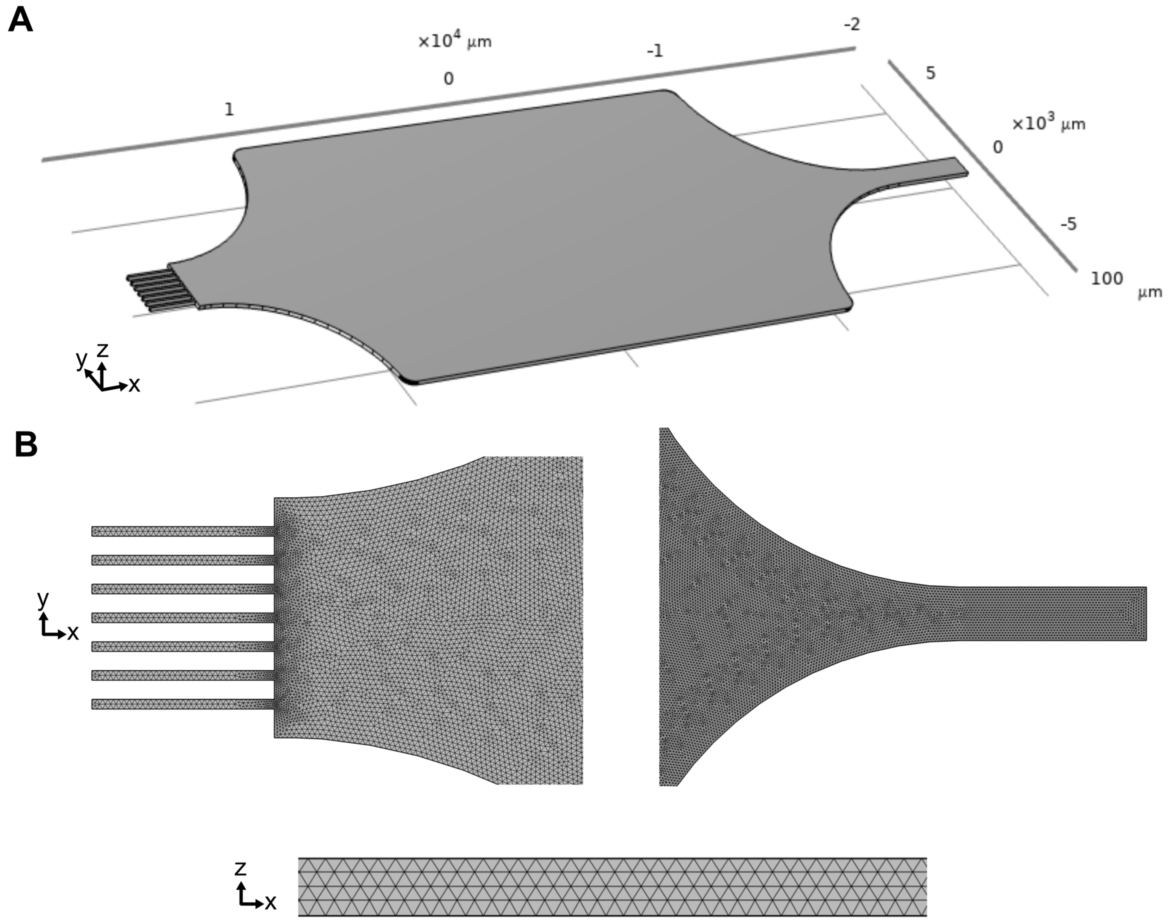

Figure S5: Computational model A) 3D microchannel geometry. B) User-controlled, unstructured mesh of the chamber near the inlets, in the cross-section in the center of chamber, and near the outlet.

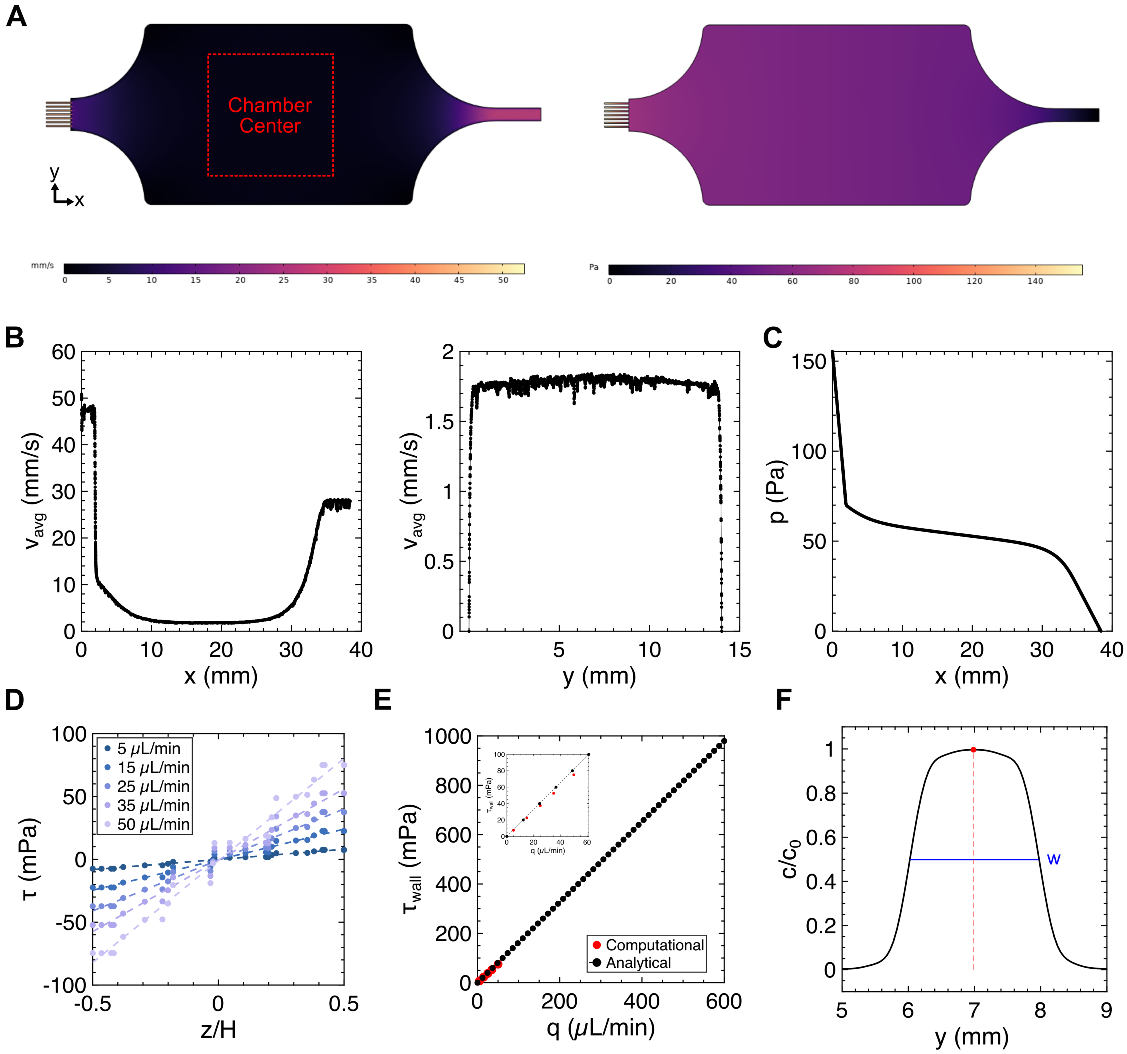

Figure S6: Simulation results A) Average velocity field and pressure field in the chamber when per-inlet flow rate is set to 25 μL min^{-1}. B) Average velocity along the axial direction and along the transverse direction of the chamber when the per-inlet flow rate is set to 25 μL min^{-1}. C) Gauge pressure along the axial direction of the chamber. The pressure drop across the microchamber is 155 Pa when the per-inlet flow rate is set to 25 μL min^{-1}. C) Shear stress, τ, profile across the channel cross-section for various per-inlet flow rates. D) Normalized concentration profile as a function of y-position with peak width at half height labeled with a blue line.

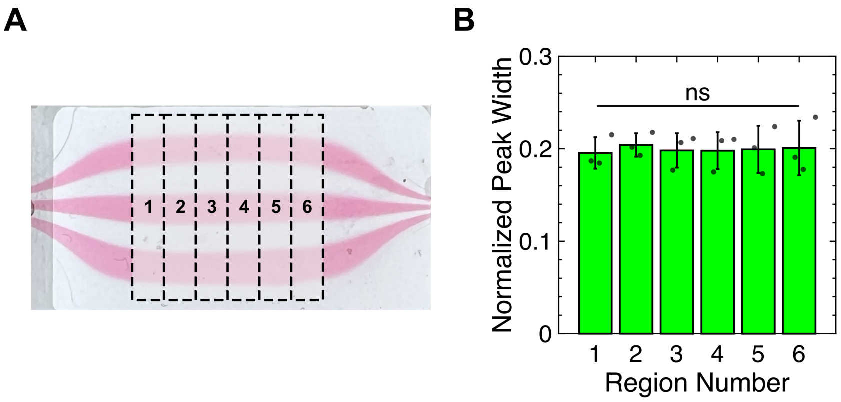

Figure S7: Concentration profile spatial uniformity at high *Pe* A) The center of the chamber is divided into six 2-mm wide regions to evaluate the variation in the stream width across the chamber width. B) Normalized intensity profiles (per-inlet flow rate of 25 µL/min) were fitted with a three-peak Gaussian model. Average peak widths (FWHM) were calculated across the regions and compared using a one-way ANOVA followed by a Tukey HSD posthoc test ($p = 0.99$).

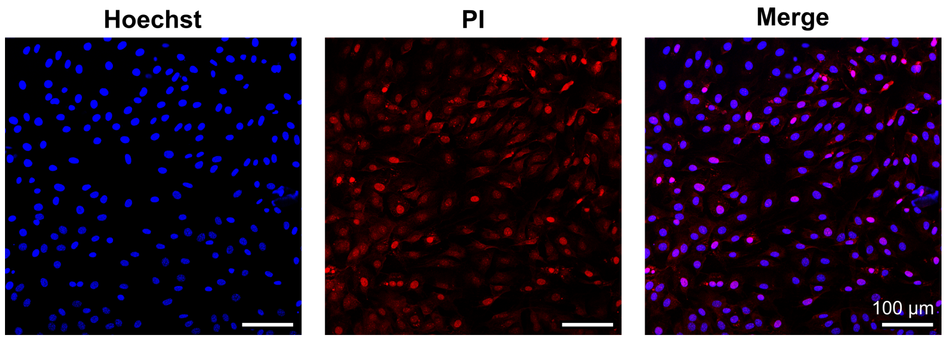

Figure S8: PI stain verification. Representative confocal fluorescence micrographs of cells stained for Hoechst 33342 (blue) and propidium iodide (PI) (red) after exposure to 30% DMSO in static conditions for 30 minutes.

Table S1: Comparison of the capabilities of previously reported reversibly sealable microfluidic gradient generators and the platform presented in this work.

Sealing Method	Maximum Pressure (kPa)	Maximum Flow Rate (μL/min)	Shear Stress (mPa)	Gradient Generator Type	Gradient Stabilization Time (min)	Chamber Area (mm^2)	Ref.
Adhesion	48	0.0567	0.45–7.2	Premixer	–	14	[1]
Adhesion	4	–	<0.1	Hydrogel membrane	120	3.5	[2]
Adhesion	–	7	–	Microjet array	45	4	[3]
Vacuum	–	1	0.43–1.4	T-sensor	–	4.8	[4]
Vacuum	4	–	0.6	Premixer	5	4	[5]
Mechanical	1.6	–	–	T-sensor	–	30	[6]
Mechanical	–	5	–	T-sensor	180	300	[7]
Mechanical	–	20	<0.1	Hydrogel membrane	15	25	[8]
Mechanical	48	4900	1.64-1000	T-sensor	1.4	400	This work

References

(1) Wang, C. J.; Li, X.; Lin, B.; Shim, S.; Ming, G.-l.; Levchenko, A. *Lab Chip* **2008**, *8*, 227–237, DOI: 10.1039/B713945D.

(2) Samandari, M.; Rafiee, L.; Alipanah, F.; Sanati-Nezhad, A.; Javanmard, S. H. *Sci Rep* **2021**, *11*, 10310, DOI: 10.1038/s41598-021-89635-0.

(3) Vit, F. F.; Nunes, R.; Wu, Y. T.; Prado Soares, M. C.; Godoi, N.; Fujiwara, E.; Carvalho, H. F.; Gaziola de la Torre, L. *Analytica Chimica Acta* **2021**, *1185*, 339068, DOI: 10.1016/j.aca.2021.339068.

(4) Chung, B. G.; Park, J. W.; Hu, J. S.; Huang, C.; Monuki, E. S.; Jeon, N. L. *BMC Biotechnology* **2007**, *7*, 60, DOI: 10.1186/1472-6750-7-60.

(5) Sip, C. G.; Bhattacharjee, N.; Folch, A. *Biomicrofluidics* **2011**, *5*, 022210, DOI: 10.1063/1.3576931.

(6) Benedetto, A.; Accetta, G.; Fujita, Y.; Charras, G. *Lab on a Chip* **2014**, *14*, 1336–1347, DOI: 10.1039/C3LC51281A.

(7) Orcheston-Findlay, L.; Hashemi, A.; Garrill, A.; Nock, V. *Microelectronic Engineering* **2018**, *195*, 107–113, DOI: 10.1016/j.mee.2018.04.011.

(8) Auxillos, J.; Crouigneau, R.; Li, Y.-F.; Dai, Y.; Stigliani, A.; Tavernaro, I.; Resch-Genger, U.; Sandelin, A.; Marie, R.; Pedersen, S. F. *Science Advances* **2024**, *10*, eadn3448, DOI: 10.1126/sciadv.adn3448.
